# A disclosure gel for visual detection of live *Bacillus anthracis* spores

**DOI:** 10.1111/jam.14226

**Published:** 2019-04-22

**Authors:** C.V. Robinson, A.H. Bishop

**Affiliations:** ^1^ Defence Science and Technology Laboratory Porton Down Salisbury Wiltshire UK; ^2^ School of Biological and Marine Sciences University of Plymouth Plymouth, Devon UK

**Keywords:** *Bacillus*, detection, enzymes, microbial contamination, spores

## Abstract

**Aims:**

To develop a gel formulation to trigger a visual signal for rapid disclosure of the location and extent of surface contamination with viable *Bacillus anthracis* spores.

**Methods and Results:**

Methylumbelliferyl‐α‐d‐glucopyranoside was combined with hyaluronic acid to produce a gel that could be applied to a surface as a coating. It remained hydrated for a sufficient time for α‐glucosidase activity present in intact *B. anthracis* spores to cleave the substrate and release the fluorescent product, methylumbelliferone. The presence of *B. anthracis* spores could be disclosed at 5 × 10^4^ CFU per reaction test well (0·32 cm^2^) both visually and using fluorescence detection equipment.

**Conclusions:**

The disclosure gel provides a rapid, visual response to the presence of *B. anthracis* spores on a surface.

**Significance and Impact of the Study:**

The disclosure gel demonstrates the first steps towards the development of a formulation that can provide nonspecialist users with a visual alert to the presence of *B. anthracis* spores on a surface. It is envisioned that such a formulation would be beneficial in scenarios where exposure to spore release is a risk, and could be used in the initial assessment of equipment to aid prioritization and localized execution of a decontamination strategy.

## Introduction

Conventional detection and identification methods following a suspected release of a bacterial agent of concern, such as *Bacillus anthracis*, include sensitive and specific genetic and antibody‐based technologies such as PCR and ELISA. Two problems encountered with such methods are surface sampling and differentiation between living and dead bacteria. Traditional plate count methods give an indication of viability but are time‐consuming and do not avoid the issue of surface sampling, which may be of particular concern given the likelihood of nonuniform agent deposition on equipment surfaces. A simple approach to detect and locate living bacteria on a surface is the exploitation of active bacterial enzymes associated with a live cell to trigger disclosure of their presence.

One feature making *B. anthracis* favourable for hostile release is its exceptional ability to survive in the environment through the formation of metabolically inert spores. However, spores must remain able to interact with their surroundings to determine an optimal time to germinate and return to a vegetative state (Moir 2006). This process does not rely on a single signal, but rather a complement of redundant germination mechanisms ensures that they will not germinate until conditions are ideal for survival (Luu *et al*. 2011).

A number of enzymes have been identified in the outermost layer of the *B. anthracis* spore; the exosporium. These include alanine racemase (Steichen *et al*. 2003) and inosine‐uridine‐preferring nucleoside hydrolase (Redmond *et al*. 2004), which interact with the environment and may function to degrade the germinants l‐alanine (Chesnokova *et al*. 2009) and inosine (Liang *et al*. 2008), respectively, to suppress germination in unfavourable conditions.

The natural course of colonization and infection of a mammal are also important stages in the lifecycle of the pathogen. To survive the onslaught of the immune system, the first‐line of defence is again through enzymes within the exosporium. Superoxide dismutase (Baillie *et al*. 2005) and arginase (Weaver *et al*. 2007) are thought to play important roles in controlling intracellular survival in macrophages through protection from oxidative injury (Cybulski *et al*. 2009) and inhibition of nitric oxide synthesis within a macrophage (Raines *et al*. 2006). The activities of superoxide dismutase, inosine hydrolase and arginase have been recorded in intact endospores (Baillie *et al*. 2005; Liang *et al*. 2008) and semi‐purified exosporium (Weaver *et al*. 2007).

This study focuses on exploiting the presence of α‐glucosidase activity, which has been detected in intact *Bacillus* spores. The general role of α‐glucosidases is to catalyse the hydrolysis of terminal, nonreducing, 1,4‐linked d‐glucose residues, with oligosaccharides being the favoured substrate. Glucosidase activity is poorly characterized in *B. anthracis*. However, it is known that α*‐* d‐glucosidase activity but not β‐d‐glucosidase activity is present in the organism (Sadler *et al*. 1984) and that in a close relative, *Bacillus cereus*, α*‐* d‐glucoside‐hydrolysing enzymes are either cytoplasmic or extracellular, depending on the availability of glucose (Suzuki and Tanaka 1981). As many synthetic colorigenic and fluorogenic substrates are unable to penetrate the cytoplasmic membrane, only secreted or membrane‐bound enzymes would allow detection of viable whole cells by this method, and thus these substrates are well‐suited to enzyme‐activated disclosure.

By combining a substrate to detect enzyme activity in viable spores with a highly hydrated polymer formulation which can easily be applied as a coating, a disclosure gel has been developed as a proof of principle concept to visually alert to the presence of *B. anthracis* endospores on a surface. The polysaccharide hyaluronic acid was selected as a carrier for the disclosure substrate due to its high moisture retention ability and viscoelasticity, combined with low immunogenicity and toxicity, which make it a potentially suitable gel for widespread use in disclosure.

## Materials and methods

### Strains, media and growth conditions

Fourteen *Bacillus* strains and 11 other common environmental or skin‐associated bacterial strains were used in this study (Table 1). *Bacillus anthracis* Sterne strain, which carries pXO1 but lacks pXO2, was used as a simulant for virulent *B. anthracis* as it can be handled in Advisory Committee on Dangerous Pathogens (ACDP) level 2 containment and has been shown to have comparable exosporium protein composition to virulent *B. anthracis* Ames (Redmond *et al*. 2004). Assay components were obtained from Sigma‐Aldrich (Gillingham, Dorset, UK) and media components from Oxoid (Basingstoke, Berkshire, UK). Nutrient agar and nutrient broth were used for general growth of non‐*Bacillus* strains. *Bacillus* strains were maintained on tryptone soy agar (TSA). The selective medium Brilliance *Bacillus cereus* agar was used to isolate bacteria of this type from soil.

**Table 1:** Bacterial strains used in this study

Bacterial species	Strain	Origin
*Bacillus anthracis*	Sterne	Dstl culture collection
*Bacillus cereus*	NCTC 8035	Dstl culture collection
*Bacillus cereus*	NCTC 9680	Dstl culture collection
*Bacillus cereus*	NCTC 9939	Dstl culture collection
*Bacillus circulans*	NCTC 2610	Dstl culture collection
*Bacillus licheniformis*	NCTC 10341	Dstl culture collection
*Bacillus megaterium*	DSM 32	Dstl culture collection
*Bacillus mojavensis*	DSM 9205	Dstl culture collection
*Bacillus subtilis*	DSM 2277	Dstl culture collection
*Bacillus subtilis*	NCTC 3610	Dstl culture collection
*Bacillus thuringiensis*	127‐S‐3	Dstl culture collection
*Bacillus thuringiensis*	186‐S‐2	Dstl culture collection
*Bacillus thuringiensis*	HD‐1 Cry^−^	Dstl culture collection
*Bacillus vallismortis*	DSM 11031	Dstl culture collection
Non*‐Bacillus* strains		
*Aeromonas hydrophila*	NCTC 8049	National Culture Type Collection (Public Health England)
*Burkholderia thailandensis*	E264	Dstl culture collection
*Escherichia coli*	MRE162	Dstl culture collection
*Micrococcus luteus*	NCTC 4819	National Culture Type Collection
*Pseudomonas fluorescens*	NCTC 10038	National Culture Type Collection
*Serratia marcescens*	NCTC 8900	National Culture Type Collection
*Staphylococcus aureus*	ATCC 29213	Dstl culture collection
*Staphylococcus aureus*	NCTC 10442	Dstl culture collection
*Staphylococcus aureus*	NCTC 33807	Dstl culture collection
*Staphylococcus epidermidis*	ATCC 12228	Dstl culture collection
*Staphylococcus epidermidis*	ATCC 14990	Dstl culture collection

### Spore preparation

Spore stocks for each *Bacillus* species were prepared by growth on NBYS (Lecadet *et al*. 1980) agar plates at 28–30°C for 4–7 days. After sporulation was verified by phase contrast microscopy, with samples containing <1% vegetative cells, the bacteria were scraped off the plates and washed six times in sterile, distilled water with centrifugation at 15 000 ***g*** for 5 min. Spore preparations were quantified through serial dilution and plating on TSA. Stocks were adjusted to 10^8^ CFU per ml with sterile, distilled water and stored at either −20°C with individual aliquots being thawed for each use (*B. anthracis* Sterne spores) or 4°C (other *Bacillus* spp.). Where dead spores were used for comparison, a spore stock at 1 × 10^8^ CFU per ml was autoclaved for 20 min at 121°C before use. Sterility was confirmed by plating 100 *μ*l of the autoclaved stock on TSA and incubating at 25°C for 3 days.

### Preparation of vegetative bacterial suspensions

Vegetative bacterial cultures were prepared by inoculation of 20 ml nutrient broth and incubation at 30 or 37°C with shaking until an OD_600_ of 0·6–0·8 was reached. The bacterial suspensions were then pelleted by centrifugation (3200 ***g*** for 5 min at 4°C) and washed three times in sterile phosphate buffered saline (PBS). Each pellet was finally resuspended in 2 ml sterile PBS and placed on ice. Vegetative bacterial suspensions were used immediately after washing. Each cell suspension was enumerated by serial dilution and plating on nutrient agar.

### Disclosure gel preparation and testing

The disclosure gel was prepared with 1 mmol l^−1^ 4‐methylumbelliferyl‐α‐d‐glucopyranoside (α‐MUG), 100 mmol l^−1^ maltose, 100 mmol l^−1^ l‐alanine, 10 mmol l^−1^ inosine and 0·7% (w/v) hyaluronic acid (mol. wt. 750 000–1 000 000) in 150 mmol l^−1^ NaCl.

Water retention of the hyaluronic acid‐based disclosure gel was measured by weight over time. Three individual 1 ml samples of the gel were pipetted onto glass slides and weighed. The slides were then incubated at 20°C, 85% relative humidity on an open tray. Weights were measured again after 3 and 6 h incubation.

To determine the effect of the formulation on germination of *B. anthracis* Sterne spores, a final concentration of 1 × 10^7^ CFU per ml spores was added to disclosure gel and incubated for 3 h at 25°C. The percentage of germinating spores, as determined by a change to phase dark, was determined through visual inspection at 400× magnification and compared to that of spores with germinants (100 mmol l^−1^ l‐alanine and 10 mmol l^−1^ inosine) alone added.

### Determination of α‐glucosidase activity using the disclosure gel

Five‐microlitres of spore stock (at 1 × 10^8^ CFU per ml) or washed vegetative bacterial suspension was added to 45 *μ*l of the disclosure gel in a flat‐bottomed black 96‐well microplate (Nunc^TM^, Fisher Scientific, Loughborough, UK). Assay reactions were incubated at 25°C for 3 h unless otherwise stated. To stop the reaction and obtain optimal fluorescence, 50 *μ*l of 0·5 mol l^−1^ NaOH was added to each well. Fluorescence was measured at excitation/emission wavelengths of 365/455 nm, using an Infinite M200 Pro fluorescence microplate reader (Tecan, Männedorf, Switzerland). Wells without spores (blank) and with autoclaved (dead) spores were used as controls. Relative fluorescence was calculated by subtracting the fluorescence level of the blank (reagents only) wells from the test wells. Each plate included a calibration curve of 4‐methylumbelliferone (4‐MU) in DMSO, ranging from 0 to 50 pmol in a total volume of 100 *μ*l, prepared in duplicate. The amount of 4‐MU released in each test well was calculated using the slope of the calibration curve. Visual observation of the fluorescence output was carried out under a UV light source with a 485–655 nm emission filter.

### Testing α‐glucosidase activity in spores dried onto a surface

Where spores dried onto the plate surface were to be tested, 5 *μ*l spore stock was pipetted into individual wells and the plates were left uncovered in a microbiological cabinet overnight. Disclosure gel was added on top of the dried spores and incubated for 3 h at 25°C before the addition of 0·5 mol l^−1^ NaOH and recording of fluorescence.

### Testing the effects of soil interference on measurement of α‐glucosidase activity

Garden soil (lime‐rich loamy soil) was suspended in sterile PBS to a concentration of 10 mg ml^−1^. Tenfold serial dilutions of the soil suspension were made both with and without the addition of *B. anthracis* spore stock (1 × 10^8^ CFU per ml final concentration) and 5 *μ*l of each dilution was tested for fluorescence output after 3 h incubation with disclosure gel. Sterile PBS was used as a negative control sample.

### Testing the effects of skin contact interference on measurement of α‐glucosidase activity

A pilot study was carried out to assess the possibility of interference with fluorescence output from the gel as a result of prior skin contact on a surface. This was crudely tested using polystyrene 6‐well plates (Corning^®^, Sigma‐Aldrich). Two individuals using unwashed hands touched the surface of each well within two 6‐well plates multiple times for 5 min. To one plate that had been handled by each individual, *B. anthracis* spore stock at 1 × 10^9^ CFU per ml was added in five drops, 2 *μ*l each, around the surface of each well. The other plate handled by each individual had sterile water added in the same manner. Sterile, untouched plates were inoculated in the same way with spore stock or water for use as comparative controls. All plates had 990 *μ*l disclosure gel added to the centre of each well, which spread to cover the surface. After 3 h incubation at 25°C and addition of 1 ml NaOH (0·5 mol l^−1^) to each well, a 100 *μ*l sample was transferred from each well into a black 96‐well plate before fluorescence was measured. Statistical analysis was carried out using Student's *t* tests with the null hypothesis being that there would be no difference between assays carried out in the presence of soil or skin contact and those carried out in their absence. Differences with *P* values of <0·05 were considered statistically significant.

## Results

### Formulation development, water retention and germination support

A range of hyaluronic acid concentrations were tested for optimal rheological properties. To evaluate the effect of various inducers and ions on α‐glucosidase activity from intact *B. anthracis* spores, a range of buffers and sugars were tested by inclusion in the formulation. Enzyme activity was also tested over a pH range of 5–7·4 to determine the optimal pH conditions. Fluorescence output was reduced in the presence of PBS, acetate buffer and phosphate citrate buffer (all pH 7) compared with 150 mmol l^−1^ NaCl; and a pH of 7–7·4 was found to be optimal for each buffer. Maltose, and to a lesser extent, sucrose, improved fluorescence output from spores, up to a final concentration of 100 mmol l^−1^ sugar (data not shown). Maltose was therefore included in the formulation as an inducer of α‐glucosidase activity. Water retention by the disclosure gel after 3 h incubation was 86 ± 0·2% and after 6 h was 69 ± 0·3%. The rate of germination‐initiation for *B. anthracis* spores in the disclosure gel was 98% but no outgrowth was observed.

### Spore‐associated α‐glucosidase activity

**Figure 1:**
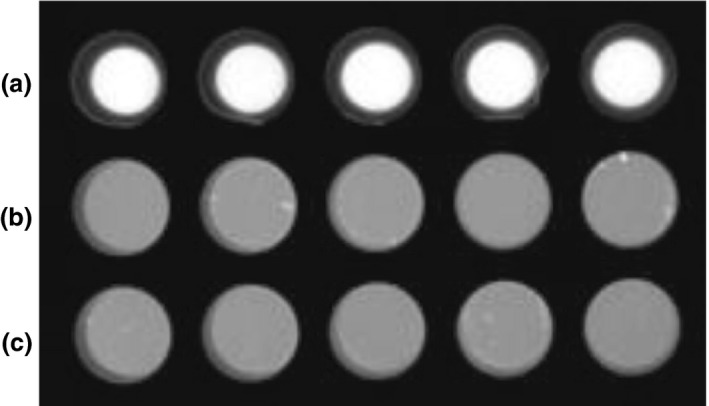
Fluorescence from disclosure gel after 3 h incubation with intact washed spores at 1 × 10^7^ CFU per ml (a), autoclaved spores at 1 × 10^7^ CFU per ml (b) and no spores (c). Fluorescence was visualized after the addition of NaOH (250 mmol l^−1^ final concentration) under UV light with a 485–655 nm emission filter.

Enzyme activity associated with intact, washed *B. anthracis* spores was measured by the detection of the fluorescent product 4‐MU released during a 3 h incubation period in the presence of α‐MUG. This method demonstrated visually detectable enzyme activity from intact spores against a background of dead (autoclaved) spores (Fig. 1). The level of fluorescent output continued to increase beyond the 3 h incubation used in the assay, so greater sensitivity could be achieved with longer incubation (data not shown). However, the given timeframe was chosen as a suitable window to achieve sufficient disclosure sensitivity whilst ensuring a reasonably rapid response could be obtained.

### Effect of nutrient germinants and growth medium on α‐glucosidase activity

The addition of freshly prepared nutrient germinants (100 mmol l^−1^ l‐alanine and 10 mmol l^−1^ inosine) led to an increase in relative fluorescence (RFU) compared to the formulation without germinants for spores at a final concentration of 1 × 10^7^ CFU per ml. From the eight experiments, the average increase was 62% with a standard deviation of ±6%. The increase in α‐glucosidase activity may be due to new enzyme synthesis as a result of initiating germination, as seen previously (Chandrapati and Woodson 2003) or release of α‐glucosidases from within the inner layers of the spore (Setlow *et al*. 2016).

### Detection of low levels of *B. anthracis* spores

**Figure 2:**
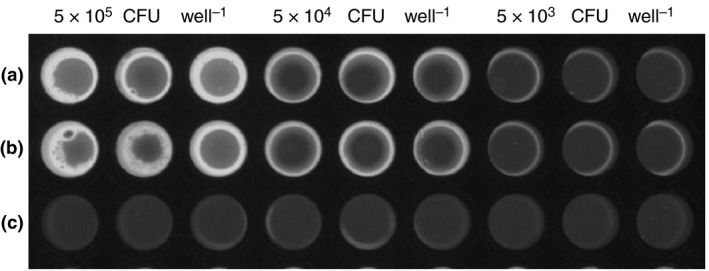
Visual fluorescence output from washed intact *Bacillus anthracis* Sterne spores in suspension (a), after drying onto the well surface (b) and autoclaved spores (c) after 3 h at 25°C. Fluorescence was visualized after addition of NaOH (250 mmol l^−1^ final concentration) under UV light with a 485–655 nm emission filter.

For spores both in suspension and dried onto a surface (0·32 cm^2^ area), 5 × 10^4^ CFU could be consistently detected above background fluorescence both using fluorescence detection equipment (Table 2) and visually under UV light (Fig. 2). Visual observation of a fluorescent output from the disclosure gel could also be made where spores were present on a surface using a hand‐held longwave UV lamp and only minimal shade from fluorescent laboratory lights. Spores that had been left to dry on a surface before the addition of the gel produced slightly lower average fluorescence output than spores in suspension, which would be expected due to lower accessibility to the substrate. Autoclaved spores could not be distinguished from background fluorescence.

**Table 2:** Relative fluorescence output from *Bacillus anthracis* Sterne spores in suspension or dried onto a surface after incubation with disclosure gel for 3 h at 25°C. RFU equates to average fluorescence output above the disclosure gel background fluorescence from eight individual experiments. Standard deviation from the mean is shown in parentheses

Spore count (CFU per well)	RFU	
	Suspension	Dried
5 × 10^5^	37 378 (±3482)	34 049 (±4042)
5 × 10^4^	1164 (±303)	608 (±311)
5 × 10^3^	0·17 (±109)	−706 (±675)
5 × 10^5^ Dead	0·00 (±58)	−2032 (±258)

### Bacterial exclusivity of the disclosure formulation

The disclosure gel was tested against a panel of *Bacillus* species and other environmental or skin‐associated bacterial species to determine its exclusivity. As expected, very little α‐glucosidase activity was detected from the intact vegetative cells tested. The substrate α‐MUG is unable to cross into the cytoplasm, which is where most known α‐glucosidases are present. The absence of medium to support bacterial growth would also have reduced the incidence of any extracellular α‐glucosidases being produced. *Bacillus thuringiensis* HD‐1 Cry^−^ had a similar level of α‐glucosidase activity to *B. anthracis* in the disclosure gel but the other environmental *B. thuringiensis* strains tested did not (Table 3). *Bacillus licheniformis* spores also displayed notable α‐MUG hydrolysis, albeit at a lower rate than both *B. anthracis* and *B. thuringiensis* HD‐1 Cry^−^. Although a spore‐associated α‐glucosidase has not been described in the literature, *B. licheniformis* is known to produce both cytoplasmic and extracellular α‐glucosidases (Thirunavukkarasu and Priest 1984) so this activity is not unexpected.

**Table 3:** Relative α‐glucosidase activity in washed spores (*Bacillus* sp.) or vegetative cells (non‐*Bacillus* sp.) after incubation with disclosure gel for 3 h at 25°C

Bacterial species	Strain	α‐glucosidase activity*	SD†
***Bacillus* strains**			
*Bacillus anthracis*	Sterne	51·1	3·7
*Bacillus cereus*	NCTC 8035	7·2	1·2
*Bacillus cereus*	NCTC 9680	2·7	0·2
*Bacillus cereus*	NCTC 9939	13·2	1·8
*Bacillus circulans*	NCTC 2610	3·1	0·4
*Bacillus licheniformis*	NCTC 10341	20·3	1·4
*Bacillus megaterium*	DSM 32	7·0	1·1
*Bacillus mojavensis*	DSM 9205	2·8	0·4
*Bacillus subtilis*	DSM 2277	0·0	0·1
*Bacillus subtilis*	NCTC 3610	6·0	0·2
*Bacillus thuringiensis*	127‐S‐3	8·1	1·5
*Bacillus thuringiensis*	186‐S‐2	9·6	1·3
*Bacillus thuringiensis*	HD‐1 Cry^−^	43·0	7·8
*Bacillus vallismortis*	DSM 11031	0·0	0·0
**Non*‐Bacillus* strains**			
*Aeromonas hydrophila*	NCTC 8049	0·1	0·1
*Burkholderia thailandensis*	E264	0·0	0·0
*Escherichia coli*	MRE162	0·3	0·0
*Micrococcus luteus*	NCTC 4819	0·0	0·0
*Pseudomonas fluorescens*	NCTC 10038	0·0	0·0
*Serratia marcescens*	NCTC 8900	0·3	0·0
*Staphylococcus aureus*	ATCC 29213	0·4	0·0
*Staphylococcus aureus*	NCTC 10442	0·0	0·0
*Staphylococcus aureus*	NCTC 33807	1·1	0·1
*Staphylococcus epidermidis*	ATCC 12228	0·74	0·1
*Staphylococcus epidermidis*	ATCC 14990	0·1	0·0

* Average amount of MU released (amol per CFU per h) from eight experiments.

† SD denotes standard deviation from the mean.

### Effect of soil and skin contamination on α‐glucosidase activity

Soil and skin contact were regarded as likely contaminants of surfaces suspected to have been exposed to bacterial agents of concern. They were, therefore, assessed for their potential interference on α‐glucosidase activity from *B. anthracis* spores using the disclosure gel.

**Figure 3:**
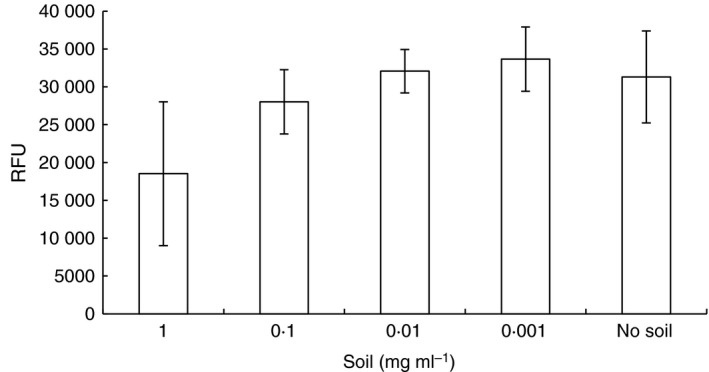
Effect of soil on observation of fluorescence. Each reaction contained 5 × 10^5^ CFU *Bacillus anthracis* spores in addition to soil suspension in PBS or PBS alone (no soil) and disclosure gel in a total volume of 50 *μ*l. RFU equates to average fluorescence output above the disclosure gel background fluorescence after 3 h incubation in 16 experiments. Error bars show standard deviation from the mean.

Garden soil suspension at a final concentration of 1 mg ml^−1^ in the gel showed considerable interference with fluorescence output, producing a significantly lower average RFU than the ‘no soil’ control (*P* = 0·006), with wide variation (Fig. 3). No difference was observed between the other soil dilutions and the ‘no soil’ control. The soil suspension was confirmed as pH 7. The interference with fluorescence may therefore be due to the organic matter present within the soil, although further investigation would be necessary to confirm this.

As an indication of the levels of *B. cereus* or *B. thuringiensis* naturally occurring in the soil sample, the selective medium Brilliance Bacillus cereus agar was used to spread culture aliquots of the soil suspension at 10 mg ml^−1^. An average of 140 blue colonies was observed per 1 ml of soil suspension, equating to fewer than one CFU per reaction well in the assay set‐up. As expected from this result, no α‐MUG hydrolysis was observed from soil suspension alone in the disclosure gel at any concentration (data not shown), suggesting that false‐positive responses to disclosure gel application would not be caused by soil contamination with this soil type.

Skin contact on a surface prior to contamination with spores and application of disclosure gel resulted in a statistically significant reduction in fluorescence (*P *≤ 0·0001). The fluorescence output was reduced by almost half over the course of the 3 h incubation (Table 4). A small but not statistically significant (*P* = 0·08) amount of fluorescence was observed on surfaces that had been handled but not exposed to *B. anthracis* spores. This is not sufficient to pose a risk of a visual false positive reaction from the gel.

**Table 4:** Fluorescence from disclosure gel in plates that had or had not been handled for 5 min, with and without the addition of 1 × 10^7^ CFU per ml *Bacillus anthracis* spores. The results were obtained from six replicate experiments. Standard deviation from the mean is shown in parentheses

	RFU	
	Touched surface	Untouched surface
+ Spores	19 788 (±3213)	37 723 (±4157)
− Spores	124 (±102)	0·0 (±12)

## Discussion

A polymer‐based gel formulation has been developed to demonstrate the first steps towards visual determination of the presence and location of surface contamination with live *B. anthracis* spores. The results show that α‐glucosidase activity present in intact *B. anthracis* spores can be used to cleave the synthetic substrate α‐MUG to produce a fluorescent response which can be visualized under UV light within a few hours.

Initial evaluation of the gel has shown that it does not respond to a range of common environmental *Bacillus* or other bacterial species under the given conditions, affording a good degree of specificity for initial assessment of equipment following a suspected release of biological agent. This would allow a more directed approach for sampling and deployment of highly specific and sensitive methods such as PCR. A number of strains tested are known to produce α‐glucosidases; in other *Bacillus* species α‐glucosidases have largely been reported in cytoplasmic or extracellular locations (Suzuki and Tanaka 1981; Thirunavukkarasu and Priest 1984; Castro *et al*. 1995) and *Staphylococcus aureus* (MalA) and *Pseudomonas fluorescens* produce cytoplasmic α‐glucosidases (Guffanti and Corpe 1975). These and most other strains tested produced little or no fluorescence in response to the disclosure gel. Therefore, their presence on the surfaces is unlikely to interfere with the visual output of the formulation or produce false‐positive signals unless present at very high inoculum levels, in the case of some *Bacillus* sp. spores, or in an active growth stage due to alternative nutrient sources; the lack of essential nutrients in the disclosure gel ensures it is not conducive to active metabolism and undesirable cell growth. Exceptions to this are some strains of *B. thuringiensis* and *B. licheniformis*, which had notable α‐glucosidase activity in their respective intact spores, although at a lower level than *B. anthracis*. This cross‐reactivity with some other *Bacillus* spp. could lead to false‐positive disclosure, therefore further work to differentiate between *Bacillus* spp. would be beneficial to optimization.

Preliminary assessments of the disclosure gel in the presence of substances that may interfere with either glucosidase activity or fluorescence output suggest that further stabilization of the gel may be beneficial for producing consistently reliable results in field environments. Soil showed a negative effect on fluorescence output only at the highest concentration tested (1 mg ml^−1^). However, the results showed huge variation between samples, which were not seen with lower concentrations of soil. The heterogeneity of the soil may be responsible for the variation seen in the fluorescence output, as it was untreated and not sieved before use. An interesting comparison would be made from testing a variety of soil types for their effects on the output from the disclosure gel. We would predict that soils with lower organic matter content would have a lesser interference effect on fluorescence.

Interestingly, *B. anthracis* spores applied to surfaces that had undergone considerable handling showed notably reduced fluorescence output from the disclosure gel. Secretions from sweat and sebaceous glands may be responsible for interference with fluorescence of the 4‐MU released from hydrolysis of α‐MUG within the gel. Further experiments will show whether this is the case or if the lowered fluorescence is a result of reduced α‐glucosidase activity. The implications of the observed interference from both soil and skin contact could lead to a reduction in sensitivity of the disclosure gel in field situations. Studies into the locality, mechanism and purpose of observed α‐glucosidase activity in intact spores have not been carried out and analyses of *B. anthracis* exosporium proteins have not identified any putative α‐glucosidases (Steichen *et al*. 2003; Redmond *et al*. 2004). It may be that the α‐glucosidase is simply a cytoplasmic enzyme in the mother cell that becomes trapped in the outer layers of the spore before its release. However, Albert *et al*. (1998) described spore coat‐associated α‐glucosidase present in *Geobacillus stearothermophilus* spores, which was supported by Setlow *et al*. (2016). The authors predicted that this enzyme may have a role in early germination and outgrowth, which may also be the case for *B. anthracis*.

Future work will address increasing the sensitivity of the gel and assessing the potential benefits of adding buffering and stabilization components to the gel to improve its reliability in a field environment. Additionally, a number of factors would need to be assessed and addressed to move this approach towards a field‐deployable solution. Conditions more closely mimicking a range of natural environments and scenarios would be needed to assess the performance of the gel in terms of maintenance of hydration, photo degradation of the fluorescent product 4‐MU and possible effects of other contaminants such as residual growth medium. The effect of the gel on equipment and what the clean‐up steps would be following disclosure are also important considerations.

## Conflict of Interest

No conflict of interest declared.
